# ALZHEIMER’S DISEASE: GENDER AND GENERATION DISPARITIES IN ATTITUDES AND HELP-SEEKING AMONG AMERICAN INDIAN ADULTS

**DOI:** 10.1093/geroni/igae098.3148

**Published:** 2024-12-31

**Authors:** Yeon-Shim Lee, Soonhee Roh, Heehyul Moon, Donald K Warne, Serene Thin-Elk, Sasheen T Stone

**Affiliations:** San Francisco State University, San Francisco, California, United States; University of South Dakota, Sioux Falls, South Dakota, United States; University of Louisville, Louisville, Kentucky, United States; Johns Hopkins University, Baltimore, Maryland, United States; South Dakota Urban Indian Health, Sioux Falls, South Dakota, United States; Yankton Sioux Tribe, Wagner, South Dakota, United States

## Abstract

**Purpose:**

While the benefits of knowledge about health and preventive measures are widely acknowledged, little attention has been paid to the knowledge and attitudes of American Indian adults towards Alzheimer’s Disease and other dementias (ADRD). Therefore, this study examines the attitudes towards Alzheimer’s Disease and help-seeking intentions among American Indian adults, with a specific focus on gender differences. Method: Convenience sampling was used to recruit 227 American Indian adults in the Northern Plains. We employed t-tests and chi-square tests to examine the differences in attitudes toward ADRD (including stigma, knowledge, and attitude) and help-seeking intentions by gender and generation (Millennial or Younger vs. Gen X or older).

**Results:**

More than two-thirds of the participants showed signs of cognitive impairment. Among the respondents, 60% were female, and 54% had completed education beyond high school. Approximately 50% were from the Millennial or younger generation. Female American Indian adults were more likely to report higher levels of ADRD knowledge, positive attitudes toward ADRD, and help-seeking intentions compared to their male counterparts. No significant differences were found in attitudes and help-seeking intentions by generation.

**Conclusion:**

The findings underscore the gender disparities in ADRD-related attitudes and help-seeking intentions among American Indian adults. Tailored educational support programs should account for these variations, focusing on strategies to encourage American Indian adults to seek an ADRD diagnosis.

